# Homocysteine Aggravates Cortical Neural Cell Injury through Neuronal Autophagy Overactivation following Rat Cerebral Ischemia-Reperfusion

**DOI:** 10.3390/ijms17081196

**Published:** 2016-07-23

**Authors:** Yaqian Zhao, Guowei Huang, Shuang Chen, Yun Gou, Zhiping Dong, Xumei Zhang

**Affiliations:** Department of Nutrition and Food Science, School of Public Health, Tianjin Medical University, Tianjin 30070, China; zyq0926@126.com (Y.Z.); huangguowei@tmu.edu.cn (G.H.); chenshuang050109@126.com (S.C.); gou.yun1990@163.com (Y.G.); dongjulia@163.com (Z.D.)

**Keywords:** homocysteine, autophagy, reperfusion injury, neurons, oxidative stress

## Abstract

Elevated homocysteine (Hcy) levels have been reported to be involved in neurotoxicity after ischemic stroke. However, the underlying mechanisms remain incompletely understood to date. In the current study, we hypothesized that neuronal autophagy activation may be involved in the toxic effect of Hcy on cortical neurons following cerebral ischemia. Brain cell injury was determined by hematoxylin-eosin (HE) staining and TdT-mediated dUTP Nick-End Labeling (TUNEL) staining. The level and localization of autophagy were detected by transmission electron microscopy, western blot and immunofluorescence double labeling. The oxidative DNA damage was revealed by immunofluorescence of 8-Hydroxy-2′-deoxyguanosine (8-OHdG). Hcy treatment aggravated neuronal cell death, significantly increased the formation of autophagosomes and the expression of LC3B and Beclin-1 in the brain cortex after middle cerebral artery occlusion-reperfusion (MCAO). Immunofluorescence analysis of LC3B and Beclin-1 distribution indicated that their expression occurred mainly in neurons (NeuN-positive) and hardly in astrocytes (GFAP-positive). 8-OHdG expression was also increased in the ischemic cortex of Hcy-treated animals. Conversely, LC3B and Beclin-1 overexpression and autophagosome accumulation caused by Hcy were partially blocked by the autophagy inhibitor 3-methyladenine (3-MA). Hcy administration enhanced neuronal autophagy, which contributes to cell death following cerebral ischemia. The oxidative damage-mediated autophagy may be a molecular mechanism underlying neuronal cell toxicity of elevated Hcy level.

## 1. Introduction

Stroke is one of the world’s leading causes of death and disability. Ischemic stroke, which accounts for approximately 85% of all strokes, occurs when there is an acute blockage of arterial blood flow to the brain tissue [1]. Homocysteine (Hcy), a sulphur-containing amino acid, does not appear to have any inherent function in the body except as a part of the methionine pathway [2]. However, it is known to have various toxic effects in the body, so any accumulation can be detrimental. The accumulation of Hcy not only increases stroke incidence but also provokes neurological deficit after stroke [3,4]. Elevated Hcy levels directly augment brain damage and induce neuronal cell death after cerebral ischemia/reperfusion [5]. It has been reported that the underlying molecular mechanisms for the neurotoxic effects of Hcy may involve the excessive activation of glutamate receptors, a rise in free calcium concentration, the disruption of DNA and the generation of reactive oxygen species (ROS) [6,7,8]. Despite this valuable study evidence, the understanding of Hcy toxicity remains incomplete. Elucidating the link between Hcy and brain cell injury is vital for improving the prevention and treatment of homocysteine-related central nervous system (CNS) disorders.

Autophagy is a highly regulated process that breaks down organelles and macromolecules through lysosomal degradation. Recently, it was shown that autophagy also plays an important role in regulating neuronal survival or death in cerebral ischemia [9]. However, whether the autophagy is cell-protective or cell-destructive is still under debate. Carloni et al. indicated that activation of autophagy protects neurons from ischemia-induced death [10], whereas another study indicated that the excessive autophagy contributes to neuron damage or death in cerebral ischemia with increased levels of light chain 3 (LC3)-II and Beclin-1 [11]. In addition to the dual role, the possible regulation of autophagic cell death after cerebral ischemia is also largely unknown. It is widely accepted that accumulation of ROS induces autophagy, and that autophagy, in turn, serves to reduce ROS levels. On the other hand, one of homocysteine’s main mechanisms of neurotoxicity is oxidative stress, which involves the formation of ROS [12]. Therefore, it is possible that ROS-mediated autophagy may be related to the neurotoxicity of Hcy in ischemia brain. Furthermore, in cardiomyocytes, Hcy has been reported to activate mitochondrial autophagy through cardiac-specific NMDA-R1 receptor [13]. There is currently no information concerning a possible association between Hcy and autophagy in ischemia brain cells. Therefore, we hypothesize that the changes in autophagy level may be involved in Hcy-induced brain damage after ischemia stroke, and ROS may be a possible link between Hcy and autophagy. 

The present study was designed to elucidate the neurotoxic effect of Hcy on ischemia brain and to determine its underlying mechanisms. Here, we provide evidence that Hcy-triggered autophagy contributes to brain neuronal cell injury and show it was accompanied by oxidative damage.

## 2. Results

### 2.1. Serum Hcy Concentration of Rats

The treatment of Hcy for 3 wk resulted in a notably higher plasma Hcy concentration in middle cerebral artery occlusion-reperfusion + homocysteine (MCAO + HCY) group and MCAO + HCY + 3-methyladenine (3MA) group, compared with MCAO animals (*p* < 0.05). The serum Hcy level after intervention was also significantly higher than the Hcy concentration before intervention (*p* < 0.05) (Table 1).

### 2.2. Hcy-Induced Neural Cell Injury in Ischemia Brain by Observation of Pathological Morphology and Apoptosis

**Figure 1:**
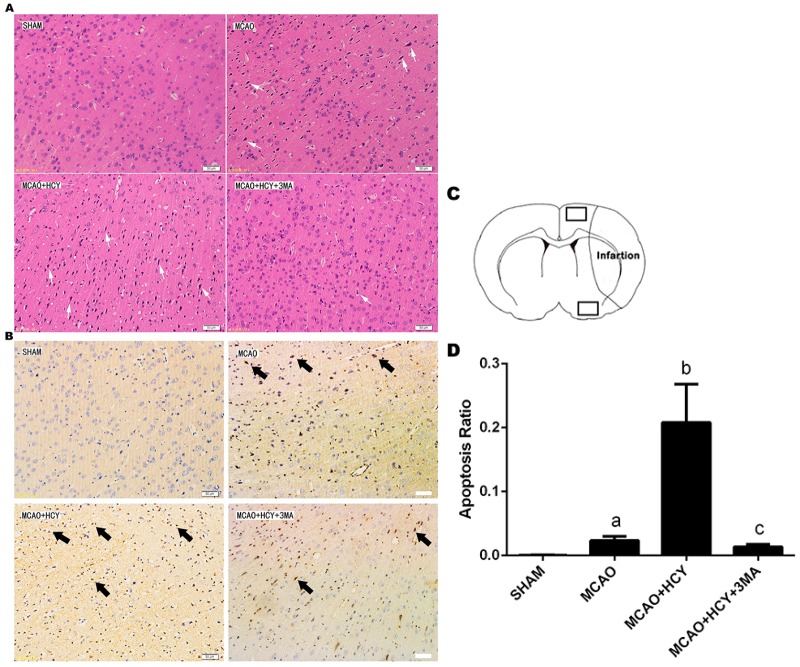
The effect of Hcy on neuronal death in ipsilateral cortex penumbra after rat focal cerebral ischemia-reperfusion. (**A**) Histologic outcomes of HE staining; (**B**) Photomicrographs of the cortex penumbra of rat brains used for the TUNEL assay. The positive cells were stained in dark brown and are indicated by arrows; (**C**) Schematic showing examples of the areas (black squares) that were selected for counting of apoptotic cells in the cortex penumbra; (**D**) Quantification of apoptotic cells by the TUNEL assay. Apoptosis was expressed as the ratio of apoptotic cells to total cells. The data are expressed as $\bar{x}$ *± s*. ^a^ *p* < 0.05 vs. SHAM group; ^b^ *p* < 0.05 vs. MCAO group; ^c^ *p* < 0.05 vs. MCAO + HCY group. *n* = 3/group. Scale bars = 50 μm.

Neuronal morphology of the rat brain was observed 72 h after MCAO by HE staining (Figure 1A). The neuronal cells in the SHAM group were arranged regularly, and the structures of neurons were clear with round, large and regular nuclei. After MCAO, most cells were arranged disorderly, with pyknotic or severely shrunken nuclei in the penumbral region. The morphology changes in the MCAO + HCY group were more severe than in the MCAO group. Compared with the MCAO + HCY group, less cellular damage was observed, and some neurons showed slightly shrunken perikarya and nuclei in the MCAO + HCY + 3MA group.

TUNEL staining was used to detect cellular apoptosis in ischemic rat brain. Apoptotic cells had typical dark brown apoptotic bodies as shown in Figure 1B,D. Compared with the SHAM group, the apoptosis rate in the brain was significantly increased following brain injury (*p* < 0.05). The increase in the apoptosis rate was more significant in the MCAO + HCY group (*p* < 0.05, vs. MCAO group). Moreover, compared with the MCAO + HCY group, the apoptosis rate was significantly decreased in the MCAO + HCY + 3MA group (*p* < 0.05). These analyses showed that Hcy could cause brain injury, whereas 3-MA partially blocked the toxic effects of Hcy on brain cells after ischemic attack. 

### 2.3. Hcy Induced Autophagosomes Accumulation and Protein Expression of LC3B and Beclin-1 in Cortex Neurons Following MCAO Injury

**Figure 2:**
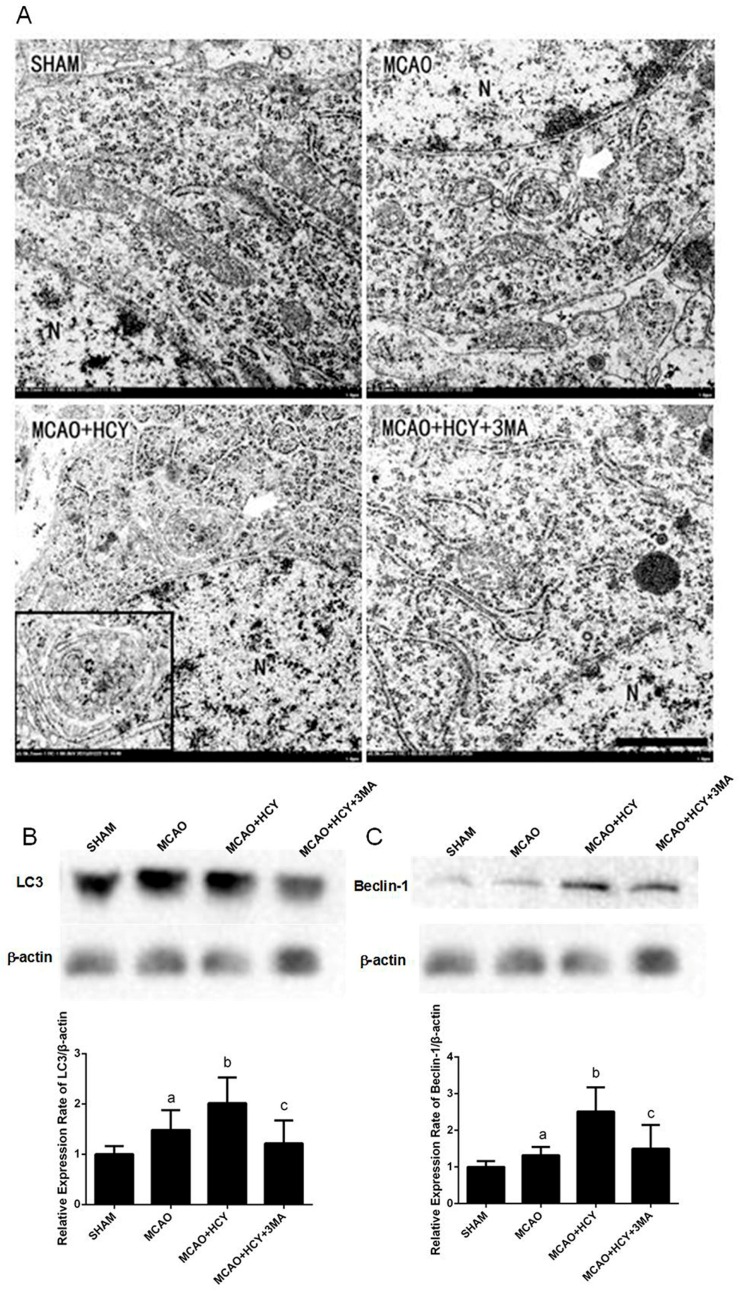
The effect of Hcy on neuronal ultrastructure and autophagic-related protein expression in the cortex after rat focal cerebral ischemia-reperfusion. (**A**) The TEM analysis of ultrastructural changes in cortical neurons 24 h after the reperfusion (*n* = 3 for each group). Arrows indicate autophagosomes. N represents the nucleus. Scale bars = 1 μm; (**B**,**C**) Representative western blots for LC3B and Beclin-1 and bar graphs show semiquantitative levels of LC3B/β-actin and Beclin-1/β-actin by band density analysis. The data are presented as the $\bar{x}$ *± s*. ^a^ *p* < 0.05 vs. SHAM group, ^b^ *p* < 0.05 vs. MCAO group, ^c^ *p* < 0.05 vs. MCAO + HCY group. *n* = 4 for each group.

Autophagy is the main pathway leading to the sequestration of the cytoplasm into the lysosome. To show whether autophagy is involved in cell death in Hcy-treated MCAO rats, transmission electron microscope (TEM) was used to directly observe the formation of autophagosomes 24 h after brain injury. As shown in Figure 2A, the TEM image of the SHAM group displayed healthy nuclei and mitochondria, abundant endoplasmic reticula, some lysosomes and many free ribosomes. In contrast, in the MCAO group, the mitochondria were visibly swollen with partially broken or disorganized cristae, the rough endoplasmic reticula developed cystic degeneration and free ribosomes decreased significantly. A few double membrane-bound compartments that contained cytoplasmic material (autophagosomes) were visible. Neuronal damage was more pronounced accompanied by more organelle lysis, and autophagosomes were frequently observed in the MCAO + HCY group than that in the MCAO group. The appearance of neurons and their organelles were less damaged, and the number of autophagosomes was also reduced in the MCAO + HCY + 3MA group compared with the MCAO + HCY group. 

To further confirm that Hcy enhanced autophagic activity in the ischemia brain, we analyzed the expression of microtubule-associated protein 1A light chain 3 (LC3B) and Beclin-1 proteins in the ischemic cortex 24 h after brain injury by western blot. Ischemia injury resulted in a significant increase in LC3B and Beclin-1 protein expression compared to the SHAM group (*p* < 0.05) (Figure 2B,C). The protein levels of LC3B and Beclin-1 were increased in the MCAO + HCY group compared with the MCAO group (*p* < 0.05). There was also a significant difference in the level of LC3B (Beclin-1) between the MCAO + HCY group and MCAO + HCY + 3MA group (*p* < 0.05). These findings further suggest that Hcy increased the protein expression of autophagy-related markers. However, 3MA treatment can reverse the effects of Hcy on autophagy in damaged brain. 

### 2.4. Hcy Induced LC3B and Beclin-1 Protein Accumulation in Neurons Not Astrocytes Following Ischemia by Immunofluorescence

**Figure 3:**
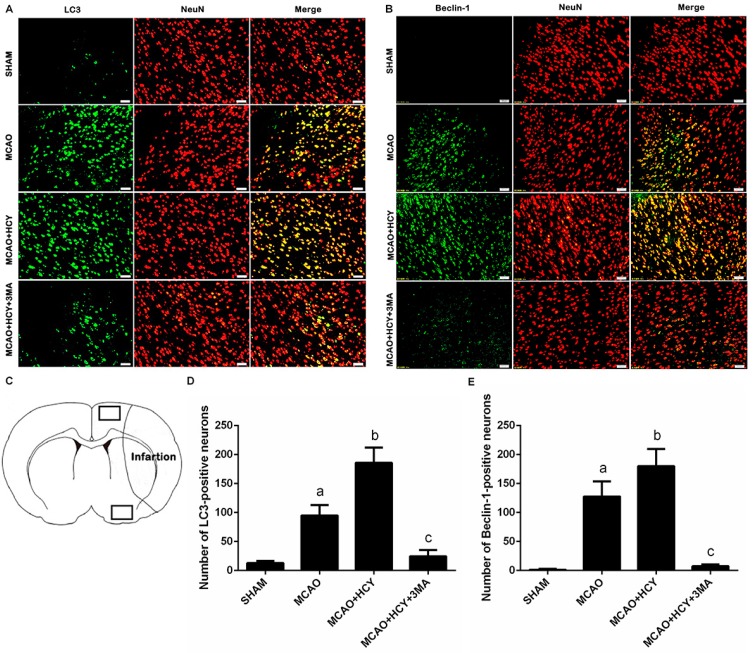
Immunofluorescence of LC3B and Beclin-1 in neurons after cerebral ischemia -reperfusion injury. (**A**,**B**) Double staining for LC3B/Beclin-1 (green) and NeuN (red) showed that increased expression of LC3B/Beclin-1 occurred mostly in cortical neurons; (**C**) Schematic showing examples of the areas (black squares) that were selected for counting of LC3B/Beclin-1 positive neurons in the ipsilateral cortex penumbra; (**D**,**E**) Quantitative assessment of LC3B/Beclin-1 positive neurons in rat brain cortex penumbra per field. Four rats in each group and 10 fields for each rat were examined. Data are expressed as $\bar{x}$ ± s. ^a^ *p* < 0.05 vs. SHAM group, ^b^ *p* < 0.05 vs. MCAO group, ^c^ *p* < 0.05 vs. MCAO + HCY group. Scale bars = 50 μm.

**Figure 4:**
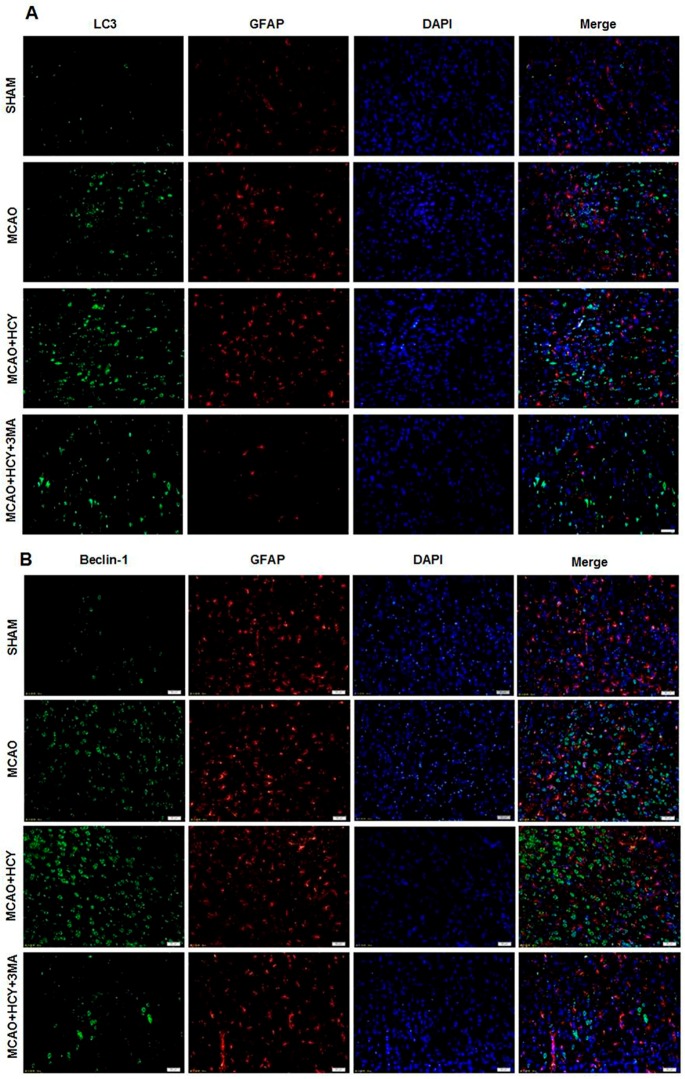
Immunofluorescence of LC3B and Beclin-1 in astrocytes after cerebral ischemia -reperfusion injury. (**A**,**B**) The analysis of the triple staining for LC3B/Beclin-1 (green), GFAP (red) and DAPI (nuclei marker, blue) of sections in the cortex indicates that LC3B/Beclin-1 was hardly expressed in astrocytes. Scale bars = 50 μm. *n* = 4/group.

To determine whether high levels of LC3B and Beclin-1 by Hcy treatment occur in a specific population of cells after cerebral ischemia, we co-stained for LC3B (or Beclin-1) and NeuN (neuron marker) (or glial fibrillary acidic protein (GFAP, astrocyte marker)). Co-staining with NeuN and LC3B (or Beclin-1) demonstrated that LC3B (or Beclin-1) was mostly present in neurons of the ischemic penumbra of cortex, and the ratio of LC3B (or Beclin-1) positive neurons and all LC3B (or Beclin-1) positive cells was close to 100% (Figure 3A,B). However, LC3B (or Beclin-1) and GFAP were not expressed in the same cell (Figure 4A,B).

Moreover, immunofluorescent staining of LC3B (or Beclin-1) and NeuN showed that the number of LC3B (or Beclin-1) positive neurons in the MCAO group was significantly higher than that in the SHAM group. There was also a marked increase in the number of LC3B (or Beclin-1) and NeuN double-positive cells in the MCAO + HCY group (vs. the MCAO group, *p* < 0.05). However, the administration of 3MA and Hcy caused a significant reduction in the number of both LC3B positive and Beclin-1 positive neurons (vs. the MCAO + HCY group, *p* < 0.05). High expression levels of LC3B and Beclin-1 in the MCAO + HCY group suggested that Hcy significantly enhanced ischemia-induced activation of neuronal autophagy. 

### 2.5. Hcy Increased 8-OHdG Protein Expression Following Ischemia

**Figure 5:**
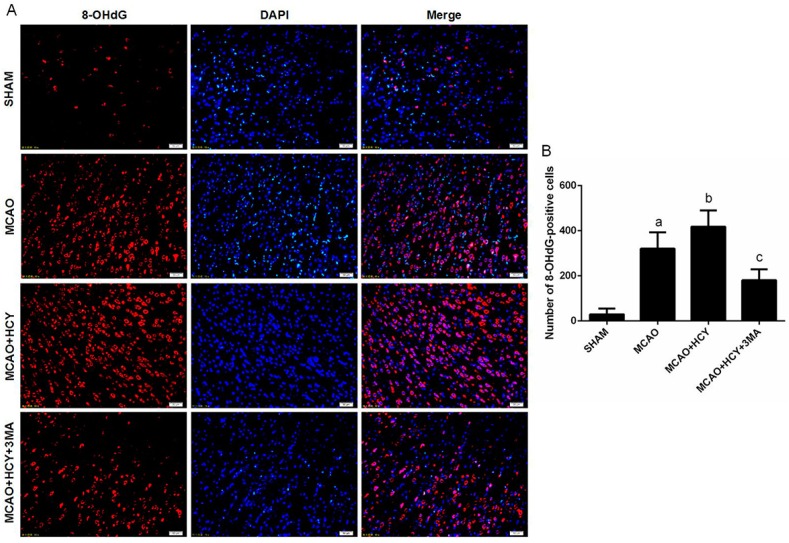
Immunofluorescence of 8-OHdG in the cortex penumbra after ischemia-reperfusion injury. (**A**) Double staining for 8-OHdG (red) and DAPI (blue) in cortex penumbra; (**B**) Quantitative assessment of 8-OHdG positive cells per field. Four rats in each group and 10 fields for each rat were examined. Data are expressed as $\bar{x}$ *± s*. ^a^ *p* < 0.05 vs. SHAM group, ^b^ *p* < 0.05 vs. MCAO group, ^c^ *p* < 0.05 vs. MCAO + HCY group. Scale bars = 50 μm.

The molecular mechanisms underlying Hcy-induced autophagy in rat brain remain to be determined. 8-OHdG is a sensitive marker of oxidative DNA damage and oxidative stress. In this study, we examined whether the injured neural cells were oxidatively stressed by a high level of Hcy using 8-OHdG as a marker. Immunofluorescent staining of 8-OHdG showed that numbers of 8-OHdG positive cells were significantly higher in the MCAO + HCY group, compared with the MCAO group. However, autophagy inhibitor 3-MA and Hcy joint intervention reduced 8-OHdG expression (Figure 5).

## 3. Discussion

As a risk factor for cerebral ischemia, homocysteine has attracted great attention, yet its neurotoxicity in the ischemia brain is unclear. In the present study, we used the model of focal ischemia to examine neural cell injury and the level of autophagy following Hcy treatment. Our results demonstrated that focal cerebral ischemia-reperfusion significantly induced brain neuronal injury. The cortex damage of the MCAO + HCY group was more severe than in the MCAO group, indicating that hyperhomocysteinemia aggravated cortex damage after ischemia-reperfusion. Hcy could also induce autophagosome accumulation, upregulate LC3B/Beclin-1 protein expression, and increase the generation of 8-OHdG. We consistently observed that autophagy stimulation by Hcy occurred mainly in cortex neurons, but not in the astrocytes with the upregulation of LC3B/Beclin-1. This study demonstrated for the first time that Hcy caused autophagy overactivation in cortical neurons following brain injury. The autophagy-promoting effects of Hcy were significantly ameliorated by inhibition of autophagy. Our results suggested that autophagy plays a crucial role in Hcy-induced injury of cells, and the oxidative damage may be involved in the mechanism.

Autophagy is an evolutionarily conserved lysosomal degradation process that serves an important mechanism for protein turnover, organelle maintenance, and the cellular stress response and is therefore essential for neuronal survival and function [14]. Previous studies in several models of cerebral ischemia (transient global ischemia [15], focal ischemia [16], and cerebral ischemia-hypoxia [17]) indicated that autophagy is involved in the ischemic brain injury, but whether it protects from or causes disease is unclear [18,19]. Autophagy activation is associated with neuroprotection in a rat model of focal cerebral ischemic preconditioning [18]. Zhang et al. showed that autophagy plays different roles in cerebral ischemia and subsequent reperfusion, and the elevated autophagy in the reperfusion phase after ischemia protects against neuronal injury by mitochondrial clearance [20]. On the other hand, it has shown that combination of ischemia and hypoxia is a powerful stimulus for autophagic lysosomal cell death in brain [21,22]. In this study, we found that the formation of autophagosomes and neuronal cell injuries were significantly increased in the brain cortex after MCAO. These effects were magnified by the increased Hcy levels. Taken together, the results suggest hyperhomocysteinemia may cause an overactivation of autophagy, which seems to be harmful for the ischemia brain. Conversely, the autophagic inhibitor 3-MA provided protection against cortical neuronal death and inhibited Hcy-enhanced autophagy after ischemia. Thus, whether autophagy is beneficial or destructive seems to depend on different types of external stimuli or the extent of autophagy, which may represent a master switch between cell death and survival after brain ischemia.

It has been reported that several key proteins govern the autophagy pathway, including Beclin-1 and LC3 [14]. Beclin-1, central to the regulation of autophagy, is involved in the formation of autophagosomes via membrane recruitment [23]. LC3 is another important molecule for autophagy as a constituent of the autophagosome membrane [24]. LC3 alone or together with Beclin-1 is often used as the marker of autophagy [25]. The enhanced expression of Beclin-1 and LC3 has been observed in brain cells after focal ischemia [22]. Consistent with the previous study, we also found that the levels of LC3B and Beclin-1 were increased after cerebral ischemia. Furthermore, we showed that Hcy treatment enhanced the autophagy with the increased levels of LC3B and Beclin-1 protein after cerebral ischemia. Double staining with NeuN (GFAP) and LC3B (Beclin-1) demonstrated that increased LC3B (Beclin-1) expression was mainly present in the neurons as opposed to the astrocytes of the ischemic penumbra of cortex. It is suggested that the elevated autophagy, which was localized primarily in neurons, may be at least partly responsible for neural injuries caused by Hcy treatment. The neurons seem to be more sensitive to Hcy than the other cell types in the brain cortex and exhibit a stronger autophagic response to Hcy stress. 

It has been extensively reported that the autophagic inhibitor 3-MA exhibits neuroprotective effects in cerebral ischemia animal models. Previous studies in several models of cerebral ischemia (permanent focal cerebral ischemia [26], cerebral ischemia/reperfusion injury [27,28]) indicated that treatment of 3-MA significantly reduced the brain infarct volume. Xing et al. reported that 3-MA treatment decreased the neuronal loss and apoptosis in the ipsilateral thalamus following focal cerebral infarction [19]. In our study, we focus on the regulatory role of Hcy in neural cell injury by autophagy, so a control group of MCAO + 3-MA could not be set alone. Despite this, it would be more helpful to elucidate the neurotoxic mechanisms of homocysteine if the MCAO + 3MA group had been set.

In addition, our data obtained with LC3 and Beclin-1 by western blot are markedly different from those observed in immunofluorescence studies. The lack of parallel increase in LC3 and Beclin-1 density in immunofluorescence and western blot levels may be due to differences in the cortical areas analyzed. For western blot, the intact cortex was separated from the rat brain, while for immunofluorescence staining, cell counting was performed in two selected cortical areas which may not represent the intact cortex. 

Cellular oxidative stress or increased generation of ROS has been reported to modulate autophagy activation in response to various stressful stimuli such as nutrient deprivation, ischemia/reperfusion and hypoxia [29,30,31,32]. For instance, an activation of autophagy contributes to neuronal cell death in neonatal rat brain exposed to hypoxia ischemia, and this is oxidative stress dependent [33]. On the other hand, oxidative stress is considered as one of the earliest events and a pathological mechanism through which hyperhomocysteinemia contributes to neurodegenerative diseases [34,35]. Oxidative damage to the cells has been reported to be associated with the autooxidation of Hcy and triggers the production of ROS [36]. The oxidized product of DNA, 8-OHdG, is the most frequently measured biomarker of the oxidative stress [37]. In this study, we found that a high Hcy level led to an increase in the 8-OHdG level and caused neural injury in the MCAO model. It is suggested that oxidative stress generation may be involved in Hcy-induced autophagy in ischemia brains. Further investigations are required to clarify the precise molecular mechanisms through which Hcy activates the oxidative stress pathway and induces autophagy in neuronal cells.

In summary, the present study demonstrated that an elevated Hcy level could enhance autophagy and aggravate neuronal cell injury following focal cerebral ischemia-reperfusion with the generation of autophagosomes and the upregulation of LC3B/Beclin-1 protein expression in neurons. Conversely, the autophagic inhibitor 3-MA could inhibit autophagy activation and ameliorate the ischemic injury caused by Hcy treatment. The oxidative stress might serve as a possible link between the overactivation of autophagy and the elevated Hcy level for ischemic stroke. Furthermore, our results supported the notion that a moderate level of autophagy may be the key for neuronal survival, while excessive induction of autophagy may aggravate cell injury or death in ischemia brain. The study may be helpful for future therapeutic efforts for autophagy-related diseases.

## 4. Materials and Methods

### 4.1. Experimental Design

Eighty male Sprague Dawley rats weighing 180–200 g (Grade SPF, Certificate Number SCXK (Jing) 20120001) were purchased from the Peking Weitonglihua Laboratory Animal Center (Beijing, China). Animal housing and application of experimental procedures were in accordance with institutional guidelines under approved protocols. All animal protocols were approved by the Institutional Animal Care and Use Committee of Tianjin Medical University (Number: TMUaMEC2012016). The rats were randomly assigned to four groups: sham operation control group (SHAM), middle cerebral artery occlusion-reperfusion group (MCAO), MCAO plus homocysteine (Sigma, St. Louis, MO, USA; 1.6 mg/kg/day) group (MCAO + HCY), and MCAO, homocysteine (1.6 mg/kg/day) plus 3-methyladenine (Sigma, 5 mmol/L, 4 mL/kg/day) group (MCAO + HCY + 3MA). Homocysteine was administered by tail vein injection for 21 days prior to SHAM or MCAO operation. The 3-methyladenine was administered by tail vein injection for 5 days prior to SHAM or MCAO operation.

### 4.2. Surgical Procedures

The rats were anesthetized with 10% chloral hydrate (3 mL/kg). Temporary focal middle cerebral artery occlusion-reperfusion (MCAO) was induced by the modified Longa method [38]. The left external carotid artery was tied up and the internal carotid artery was closed. A nylon thread was advanced through the left internal carotid artery to the origin of the middle cerebral artery (MCA). One hour after the operation, the thread was pulled out 1 cm and cut off. Rats subjected to the SHAM operation were treated similarly, while the thread was not advanced to the origin of the MCA. The animals were separately sacrificed at 24 and 72 h after reperfusion for the following experiments.

The modified Longa method was used to assess the neurological deficit [38]. A neurological score was assigned to each rat as follows: 0 = no deficit; 1 = contralateral forelimb weakness; 2 = circling to contralateral side; 3 = partial paralysis on contralateral side; and 4 = no spontaneous motor activity. Rats treated with the MCAO procedure with neurological deficit scores of 1–3 were selected for subsequent experiments.

### 4.3. Serum Homocysteine Concentration

Blood (1 mL) was taken from rats by the angular vein before intervention and the surgical operation, and centrifugalized at 3000 rpm for 10 min. The supernatant was collected. The serum Hcy was measured using a cycling enzymatic method with a commercially available kit (Nanjing Jiancheng Bioengineering Institute, Nanjing, China), and analyzed with an automatic biochemical analyzer (CS T300, Dirui Medical Technology Ltd., Changchun, China).

### 4.4. Transmission Electron Microscopy (TEM)

Transmission electron microscopy was used to identify ultrastructural changes in cortical neurons 24 h after brain injury. Fragments of the cerebral cortex were fixed with 2.5% glutaraldehyde solution, fixed with 1% osmic acid, gradient acetone dehydrated, and then the samples were embedded in an Epon/Araldite mixture. Embedded fragments were then sliced and stained with uranyl acetate and lead citrate, and viewed under a HT-7700 TEM (Hitachi, Tokyo, Japan).

### 4.5. Preparation of Paraffin Section

Rats were separately anaesthetized 24 and 72 h after reperfusion (24 h for immunofluorescence and 72 h for HE and TUNEL). Then, the rats were perfused with 0.9% saline solution followed by 4% phosphate-buffered paraformaldehyde (PFA). Afterwards, brains were removed, postfixed, equilibrated in 30% sucrose in phosphate-buffered saline and embedded in paraffin. Coronal sections at 6 μm were used for immunofluorescence, HE and TUNEL.

### 4.6. HE and TUNEL Staining

Brain cortex paraffin sections were stained with HE for routine examinations and photographed using a light microscope (IX81; Olympus, Tokyo, Japan). Apoptotic cells in brain tissue sections were identified by in situ cell death detection kit (Roche Company, Basel, Switzerland) as described previously [39]. Paraffin sections were deparaffinized, treated with 3% hydrogen peroxide and TdT-enzyme, incubated with digoxigenin-conjugated antibodies and colorized with DAB (Solarbio, Beijing, China). Sections were photographed using a light microscope (IX81; Olympus). Positive neurons were counted using Image Pro Plus 6.0 (Media Cybernetics, Silver Spring, MD, USA).

### 4.7. Immunofluorescence

Sections were de-waxed and hydrated to dispose 3% H_2_O_2_ for 10 min at room temperature, then repaired by citric acid antigen, blocked with goat serum for 40 min at 37 °C, incubated with the first antibody (LC3B (1:400; Cell Signaling Technology, Boston, MA, USA), Beclin-1 (1:200; Abcam, Cambridge, MA, USA), 8-Hydroxyguanosine (8-OHdG; 1:500; Abcam), NeuN (1:1000; Abcam) and GFAP (1:100; Abcam)) overnight at 4 °C and incubated with the goat anti-rabbit or goat anti-mouse secondary antibodies (1:100; Zhongshan Goldbridge Biotechnology, Beijing, China) for 1 h at 25 °C. A fluorescence microscope (IX81; Olympus) was used to observe and photograph. Positive cells were counted by Image Pro Plus 6.0. 

### 4.8. Western Blot

Rats were sacrificed 24 h after reperfusion. Western blot was used to analyze protein expression in the ischemic cortex. In brief, the brain tissues were homogenized in RIPA buffer (20 mmol/L TRIS-HCl pH 7.5, 150 mmol/L NaCl, 1 mmol/L EDTA, 1% Triton-X100, 0.5% sodium deoxycholate, 1 mmol/L PMSF and 10 µg/mL leupeptin), incubated on ice for 30 min, and centrifuged at 14,000× *g* for 25 min at 4 °C. The supernatants were collected, and protein concentration was detected with BSA as a standard according to the Bradford method [40]. Equal amounts of protein from each sample were separated by 10% sodium dodecyl sulfate-polyacrylamide gel electrophoresis and transferred to nitrocellulose blotting membranes (NC membranes; Millipore, Bedford, MA, USA) by the wet electrical transfer method. The membranes were then blocked with 5% milk (Sigma) in 1× TBST for 1 h at room temperature, followed by incubation with the primary antibodies (LC3B 1:1000, Beclin-1 1:500, β-actin (1:10,000; Abcam)) overnight at 4 °C and incubated with the goat anti-rabbit or goat anti-mouse secondary antibodies (1:10000; Zhongshan Goldbridge Biotechnology) for 1 h at 25 °C. Then, the blots were developed by immobilon western chemiluminescent horseradish peroxidase substrate (Millipore) and observed using a ChemiDoc^TM^ XRS+ Imaging System (Bio-RAD, Hercules, CA, USA). The protein levels were quantified by densitometry using Image J 1.4.3 Software (National Institutes of Health, Bethesda, MD, USA) and calculated according to the reference bands of β-actin. 

### 4.9. Statistical Analysis

Statistical analysis was performed using SPSS, version 19.0 (SPSS, Chicago, IL, USA). The results are presented as mean ± standard deviation ($\bar{x}$ *± s*). Differences between means were evaluated by one-way analysis of variance (ANOVA) followed by Least Significant Difference (LSD) multiple range test if data conformed to normality and homogeneity of variance, or using a non-parametric method (Kruskal–Wallis test) followed by the Mann–Whitney *U*-test using Bonferroni correction if not. *p* < 0.05 was considered to be statistically significant.

## Figures and Tables

**Table 1:** The concentration of serum Hcy before and after intervention in rats.

Groups	Before Intervention (μM)	After Intervention (μM)
SHAM	5.40 ± 2.72	5.20 ± 2.02
MCAO	7.20 ± 0.80	6.90 ± 2.47
MCAO + HCY	7.00 ± 1.77	15.07 ± 2.66 ^a,b,^*
MCAO + HCY + 3MA	6.40 ± 2.26	10.53±0.83 ^a,b,^*

Data are expressed as $\bar{x}$ *± s* (*n* = 5). ^a^ *p* < 0.05 vs. the sham operation control (SHAM) group, ^b^ *p* < 0.05 vs. MCAO group, * *p* < 0.05 vs. the concentration of Hcy before intervention.
